# The mixed epithelial stromal tumor of the kidney: A recently recognized entity

**DOI:** 10.4103/0970-1591.33737

**Published:** 2007

**Authors:** Gaurav Gupta, Santosh Kumar, Jayalakshmi Panicker, Anila Korula

**Affiliations:** Department of Urology, Christian Medical College, Vellore, Tamilnadu, India; * Department of Pathology, Christian Medical College, Vellore, Tamilnadu, India

Over the past few years, a rare distinctive kidney tumor has been described. This benign tumor contains epithelial and spindle cell stromal components and arises exclusively in women. These tumors are known as mixed epithelial stromal tumor (MEST) of the kidney. MEST is a new entity that has been included in the WHO 2004 renal tumor classification.[1] We report a case of a post- menopausal, 46-year-old lady, with a large right renal mass who presented with episodic bilateral loin pain.

## CASE REPORT

A 46-year-old, post-menopausal lady had episodic bilateral loin pain for one year. She had no associated LUTS and no history of hematuria, flushing, headache or palpitation. She was hypertensive on irregular treatment. Physical examination was unremarkable. Her routine blood investigations and urine microscopic examination was normal. Evaluation for functional adrenal tumor was negative.

**Figure 1:**
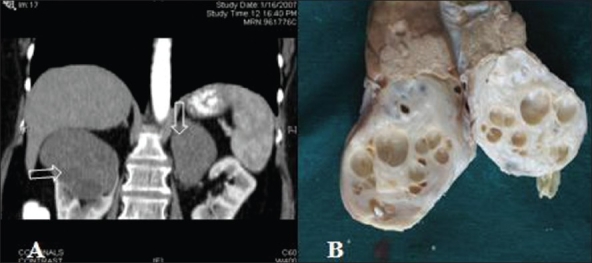
A) Contrast enhanced CT (Coronal section) shows a 75×55 mm hypodense well-defi ned, minimally enhancing mass arising from the upper pole of the right kidney. Multiple rounded fluid-density foci were noted within this mass (horizontal arrow) suggestive of areas of cystic change or necrosis with a normal right adrenal. A 4.5×6.5cm left adrenal hypodense mass was also seen (vertical arrow). B) Gross specimen shows sold and cystic areas

Contrast enhanced CT scan abdomen showed a right renal 75×55 mm hypodense well-defined, minimally enhancing mass arising from the upper pole [Figure 1A] Multiple rounded fluid-density foci were noted within this mass suggestive of areas of cystic change or necrosis with a normal right adrenal. A 45×65 mm left adrenal hypodense mass was also seen. The differential diagnosis of right renal cell carcinoma metastasizing to the left adrenal gland was made. She had a biopsy of the left adrenal, which was reported as adrenal ganglioneuroma.

**Figure 2:**
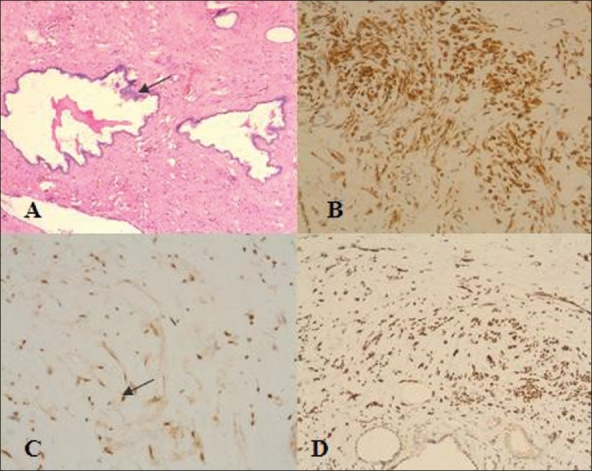
A) H & E staining (×100): Cystically dilated tubular structures lined by cuboidal epithelium with hobnailing (arrow)set in a stroma with loosely interspersed spindle cells. B) Immunostaining (×200) for muscle specificaction: The spindle cells in the stroma show cytoplasmic positivity. C) Estrogen receptor (ER) immunostaning (×400): spindle cell nuclei show positive staining for ER (arrow). D) Immunostaining for SMA (×200): spindle cells on the stroma stain for Smooth Muscle Actin (SMA)

On gross examination, the tumor was encapsulated well-circumscribed with solid and cystic areas [Figure 1B]. The adjacent renal parenchyma was unremarkable. On microscopic examination, tumor was composed of spindle to elongated cells with elongated plump nuclei and moderate amounts of eosinophilic cytoplasm. Many cystic spaces were seen lined by flattened to cuboidal cells [Figure 2A]. Occasional clusters of dysplastic and immature renal tubules were seen, some of which were cystically dilated. There were areas of hyalinization, myxoid change and mild infiltrates of lymphocytes, plasma cells and mast cells. There were fascicles of smooth muscle fibers in the stroma. Immunohistochemistry showed the tumor cells were positive for muscle actin [Figure 2B] and smooth muscle actin (SMA) [Figure 2D] and were negative for CD34 and vimentin. There was focal nuclear membrane positivity for estrogen and pogesterone receptors of the stromal cells [Figure 2C]. There were areas of stromal hyalinization and edema. There was no evidence of malignancy. Left adrenal microscopic features were typical of ganglioneuroma.

## DISCUSSION

The term mixed epithelial and stromal tumor of the kidney (MEST) was first introduced by Michal and Syrucek in 1998 to describe a biphasic cystic lesion with grossly recognizable stroma in a middle-aged woman. Radiographically, a complex cystic pattern is predominant, typically classifying as Bosniak Type III-IV lesion.[2] Though the number of MEST of the kidney has been on rise, only one case has been reported from India. To the best of our knowledge this is the first documented case of MEST in the Indian literature. Mixed epithelial stromal tumor is characterized grossly by mixture of solid and cystic areas and is microscopically composed of proliferation of epithelial and stromal cells.[3] In the past, other names, such as cystic hamartoma of the renal pelvis or adult mesoblastic nephroma, cystic nephroma, mature nephroblastic tumor and cystic partially differentiated nephroblastoma were applied to such lesions.[3]

Common clinical presentations are those of usual renal mass such as flank pain, hematuria and urinary tract infection or incidentally diagnosed . Mean tumor size reported in the literature is 6 cm and the tumor shows a female preponderance centered on peri-menopausal age and frequent reactivity for estrogen and progesterone receptors.[4] In the literature a different hypothesis has been proposed for its origin and it has been postulated that a deranged hormonal environment namely, peri-menopausal changes or therapeutic hormones with unopposed estrogen, induces the proliferation of peri-ductal fetal mesenchyme, which has the capacity for dual, mesenchymal and epithelial differentiation and presents around the epithelial structure in organs like kidney, liver and pancreas. This theory has been corroborated by the presence of estrogen and progesterone receptor expression in the spindle cell.[3,4] Others suggested embryologic hypothesis as mullerian displacement in the kidney.[3]

Differential diagnoses for MESTs are limited. Adult mesoblastic nephroma is the primary diagnosis in the majority of this group of tumors.[3] MEST shares a few morphologic characteristics of mesoblastic nephroma, but mesoblastic nephroma has frequent infiltration in the surrounding renal parenchyma or hilum which is rarely encountered with MEST.[5] However, it is now clear, based on molecular studies, that MEST has no relationship to mesoblastic nephroma.[6] MEST and cystic nephroma share overlapping morphological features. While the former has strong association with the female sex, the latter can affect both sexes and, on occasion, may also have hormonal association. The current theory is that MEST may be related to cystic nephroma and that there may be a spectrum of lesions that lie between these two entities.[3] However, further studies, including molecular studies, are needed to support this theory.

The prognosis of this tumor is favorable[3] but malignant cases have also been reported.[4] Although malignancy of the epithelial component is not yet documented in MEST, there are a few reports of stromal malignancy.[4] As for Bosniak Type III-IV cyst, radical nephrectomy is the treatment of choice. Therefore it appears that MEST has a benign course but can have aggressive behavior that requires follow-up.
